# Quantitative Sensory Testing in Adolescents with Co-Occurring Chronic Pain and Obesity: A Pilot Study

**DOI:** 10.3390/children7060055

**Published:** 2020-06-02

**Authors:** Keri R. Hainsworth, Pippa M. Simpson, Omar Ali, Jaya Varadarajan, Lynn Rusy, Steven J. Weisman

**Affiliations:** 1 Department of Anesthesiology, Medical College of Wisconsin, Milwaukee, WI 53226, USA; jvaradarajan@chw.org (J.V.); lrusy@chw.org (L.R.); sweisman@chw.org (S.J.W.); 2 Jane B. Pettit Pain and Headache Center, Children’s Wisconsin, Milwaukee, WI 53226, USA; 3 Division of Quantitative Health Sciences, Medical College of Wisconsin, Milwaukee, WI 53226, USA; psimpson@mcw.edu; 4 Department of Pediatrics, Medical College of Wisconsin, Milwaukee, WI 53226, USA; oali@valleychildrens.org; 5 Department of Endocrinology, Children’s Wisconsin, Milwaukee, WI 53226, USA

**Keywords:** chronic pain, obesity, pain threshold, quantitative sensory testing, sensory functioning

## Abstract

Factors such as gender, ethnicity, and age affect pain processing in children and adolescents with chronic pain. Although obesity has been shown to affect pain processing in adults, almost nothing is known about pediatric populations. The aim of this pilot study was to explore whether obesity alters sensory processing in adolescents with chronic pain. Participants were recruited from a chronic pain clinic (Chronic Pain (CP), *n* = 12 normal weight; Chronic Pain + Obesity (CPO), *n* = 19 overweight/obesity) and from an obesity clinic (Obesity alone (O), *n* = 14). The quantitative sensory testing protocol included assessments of thermal and mechanical pain thresholds and perceptual sensitization at two sites with little adiposity. The heat pain threshold at the hand was significantly higher in the CPO group than in either the CP or O groups. Mechanical pain threshold (foot) was significantly higher in the CPO group than the CP group. No differences were found on tests of perceptual sensitization. Correlations between experimental pain and clinical pain parameters were found for the CPO group, but not for the CP group. This preliminary study provides important lessons learned for subsequent, larger-scale studies of sensory processing for youth with co-occurring chronic pain and obesity.

## 1. Introduction

Pediatric chronic pain affects between 25%–37% of children and adolescents [1,2], and is associated with widespread negative effects on the lives of patients and their families [3], including disruptions in daily functioning [4,5] and psychological distress [6]. A growing number of studies are showing that the impact of pediatric chronic pain is exacerbated by obesity. Youth with obesity seen in a multidisciplinary pain clinic are more likely than youth with healthy weight to have impaired health-related quality of life [7] and impaired functional disability (by parent report) [8], and are more likely to wait longer for a referral to a pain clinic [9]. This is concerning, given that youth with co-occurring chronic pain and obesity do not benefit from multidisciplinary pain treatment, whereas youth with chronic pain and normal weight do [10]. While the complex, multifactorial nature of both chronic conditions make it difficult to explain the treatment failure in this population, it is plausible that somatosensory system processing is altered by obesity [11,12], which could complicate treatment response in youth with chronic pain. At present, however, as with most other non-disease-related pain conditions in children [13], we know almost nothing about the underlying pathophysiology in this population.

Quantitative sensory testing (QST) may play a key role in better understanding whether obesity alters sensory functioning in pediatric chronic pain. QST refers to a set of psychophysical methods used to assess pain sensitivity and functioning of small- (A-Delta and C-fiber) and large-caliber (A-Beta fiber) sensory nerves [14]. As an objective method to better understand pain conditions, QST is increasingly being utilized to examine the mechanisms underlying recurrent and chronic pain conditions in adults [13,15]. Importantly, QST allows for the examination of local or peripheral alterations in sensory functioning (e.g., via assessment of responses to phasic stimuli) as well as central dysfunction (e.g., via assessment of responses to tonic stimuli) [15]. 

A small, but growing number of groundbreaking pediatric studies have employed QST to examine sensory functioning in healthy children [14,15] and in youth with pain conditions. Among the latter are studies of sensory features and peripheral neuropathies in chronic regional pain syndrome [16], pain thresholds and sensitization associated with migraines [17], pain thresholds in youth with recurrent abdominal pain [18], altered sensory thresholds [19,20] and sensitization [20] in children with sickle cell disease, pressure pain thresholds in adolescents with juvenile fibromyalgia [21], and altered sensory functioning in youth with cerebral palsy [22]. In a large, population-based study, Tham et al. [23] found altered sensory functioning in youth with chronic pain, as well as relationships between clinical pain parameters and QST measures. These studies suggest that pediatric chronic pain alone can negatively affect both peripheral and central sensory processing.

Although the evidence is mixed, QST studies have shown that obesity also alters sensory functioning. For example, studies have shown that obesity is associated with lower pain thresholds (greater pain sensitivity) [24,25,26], whereas others have found obesity to be associated with higher pain thresholds (lower sensitivity) [11,27,28,29] and difficulty grading pain intensity [29]. Price et al. [12] hypothesized that subcutaneous adiposity may explain the conflicting findings. These authors tested a body site with a high degree of adiposity, the abdomen, and sites with little adiposity, including the forehead and thenar eminence of the hand. When compared against a control group with normal weight, adults with obesity had higher pain thresholds at the abdomen, but not at the other sites, and no between-group differences were found for central pain processing. Other studies, however, have not supported this hypothesis [11,30]. To our knowledge, only one pediatric QST study like this exists, and no differences were found in pressure pain thresholds for healthy participants with or without obesity [31]. Although the discrepancies across these studies currently lack a unifying explanation, the adult studies primarily suggest that obesity does alter sensory functioning. This has important implications for the treatment of pain in general [12], the management of post-surgical pain [11,29], and chronic pain treatment in those with obesity [29]. Given these implications, it is surprising that so few studies have been conducted, and almost no studies have examined sensory functioning in those with co-occurring chronic pain and obesity. To our knowledge, only two adult studies of sensory functioning (albeit without standardized QST methods) have involved participants with both conditions. Both studies [24,32] included participants with fibromyalgia and co-occurring obesity, and both reported higher pain sensitivity in those with obesity. While neither study examined underlying mechanisms, both suggest that obesity could affect peripheral and central pain processing.

The primary aim of this pilot study was to provide preliminary evidence on whether relative differences in pain thresholds and central sensitization exist between youth with chronic pain + underweight/normal weight, chronic pain + overweight, chronic pain + obesity, and a group with obesity. Both heat and mechanical pain thresholds were examined, because thermal and mechanical sensory modalities are typically used in order to increase the sensitivity of the assessments [17]. Testing is also typically done on two body sites for each sensory modality: a pain-relevant and a distal site. As there are no QST data available on chronic pain and obesity in youth, we tested the thenar eminence of the non-dominant hand, as it is the non-pain site used in most QST studies. Additionally, it is important that this site was utilized in a study on sensory functioning in adults with obesity, specifically to avoid the potential confound of excess subcutaneous adipose tissue [12]. The second site chosen was the lateral dorsum of the foot. It is a frequently used site in pediatric QST studies [15], and we reasoned that it was also an area of the body least likely to have thresholds affected by excess subcutaneous adipose tissue.

Analyses showed no differences between pain parameters or QST measures in the chronic pain + overweight and chronic pain + obesity groups; therefore, these groups were combined for this pilot. This study was designed to show differences in pain thresholds and central sensitization between the three groups. 

## 2. Materials and Methods

### 2.1. Research Design

This study utilized a cross-sectional design. Group 1 included adolescents with chronic pain and underweight/normal weight (CP) (*n* = 12); Group 2 included adolescents with chronic pain + co-occurring overweight/obesity (CPO) (*n* = 19); and Group 3 (*n* = 14) included adolescents with obesity (O). 

### 2.2. Participants

Adolescents between 13 and 17 years of age were recruited for the CP and CPO Groups from the multidisciplinary pain clinic at a pediatric hospital in the Midwest region of the United States. Youth are referred to the pain clinic by other physicians or specialized tertiary care clinics for treatment of complex chronic pain. Children of the same age range were recruited for the O Group from patients seen in the weight management clinic at the same hospital. Youth with a Body Mass Index (BMI) ≥ the 95th percentile for gender and age and at least one medical comorbidity are referred to the clinic by pediatric providers. All participants were asked to abstain from short-acting analgesics for a period of 24 h prior to testing. Exclusion criteria included a diagnosis of diabetes mellitus and current use of long-acting analgesic medications. Due to the associated analgesic properties of psychotropic medications, potential participants were also excluded if currently using anti-depressant, or anxiolytic medication(s) [33,34,35]. 

All participants who met eligibility criteria were approached for participation. All participants and their parents received a gift card to compensate them for their time. Participants gave assent, and parents gave consent. This study was approved by the children’s hospital institutional review board (#167006). 

### 2.3. Quantitative Sensory Testing

All testing was done by the primary author (with extensive training in psychophysics), and took place in the hospital’s pediatric translational research unit. The testing protocol for the current study was modeled after a protocol used in a published QST study focused on pediatric chronic pain [17]. Testing took place in a quiet room, with participants acclimating to the room’s ambient temperature (including taking off all footwear on the foot to be used in testing) for 10–15 minutes prior to testing. Both heat and mechanical sensory modalities were assessed at the thenar eminence of the non-dominant hand and at the lateral dorsum of the foot. 

All participants were tested alone. During testing, the test site (hand or foot) was hidden from the participant’s view, as was the computer screen. Additionally, no visual or verbal cues were given to indicate the start of any stimulus. All tests were conducted in the following sequence: (1) heat pain threshold (HPT): a. hand, b. foot; (2) heat perceptual sensitization (HPS): a. hand, b. foot; (3) mechanical pain threshold (MPT): a. hand, b. foot; (4) mechanical perceptual sensitization (MPS): a. hand, b. foot. The instructions used for participants were obtained from Zohsel et al. [17] and translated from German to English for use in this study. Each threshold assessment consisted of 5 trials, with each series preceded by three (heat) or by one (mechanical) test trial. Perceptual sensitization assessments were preceded by only 1 heat or 1 mechanical test trial.

#### 2.3.1. Heat Pain Threshold (HPT)

Heat stimulation was delivered by a Medoc TSA-II Neuro Sensory Analyzer (Medoc Ltd., Ramat Yishai, Israel), with a 16 × 16 mm Peltier thermode placed on the participant’s skin. A velcro strap was used to maintain skin contact. Using the method of limits, responses to stimuli were made by pushing a button at the point when participants first felt pain, at which point the stimulus was stopped and return to baseline temperature immediately followed. Each trial began at a baseline temperature of 32 °C, which increased (hot) at a rate of 1 °C/s. The inter-trial interval was at least 12 s. To ensure the safety of the testing protocol, the upper limit of the TSA-II was set to 50 °C. In cases when the participant did not report pain prior to the cutoff, 50 °C was used in the calculation of the HPT. HPT was defined as the mean of the 5 experimental trials.

#### 2.3.2. Heat Perceptual Sensitization (HPS)

The test began with the participant holding the button until his/her pain threshold was reached (T1). At that point, the stimulus was held constant for 30 s, after which participants adjusted the temperature so that it matched T1 (i.e. the pain threshold). Participants were told that they could leave the temperature unchanged, lower it, or increase it, but they were not told that the temperature did not change during the tonic stimulation. Heat perceptual sensitization for each experimental trial was calculated as the difference between the 2 temperatures (adjusted temperature - pain threshold, or ΔT = T2 – T1). As in previous studies, a negative temperature change indicated perceptual sensitization, and a positive temperature change indicated habituation. The mean of the experimental trials was calculated to determine heat perceptual sensitization (HPS).

#### 2.3.3. Mechanical Pain Threshold (MPT)

The mechanical pain threshold was assessed with 7 standardized punctuate filaments (MARSTOCK Nervtest, OptiHair_2_ Von Frey Filaments, Germany) with a blunt tip (diameter: 0.2 mm). The probes (8, 16, 32, 64, 128, 256, 512 mN) were applied to a skin area of approximately 1 cm^2^. The procedure began with the lowest intensity probe, with application to the skin in five ascending and descending series. Using the method of levels, participants were instructed to answer with a “no” for a non-painful sensation and “yes” for a sensation at the pain threshold. MPT was defined as the geometric mean of the 10 resulting values above and below the threshold. For each series in which the participant did not indicate that he/she felt a painful sensation, the extreme value was used in the calculation of the threshold. 

#### 2.3.4. Mechanical Perceptual Sensitization (MPS)

The von Frey filament closest to the mechanical pain threshold was used to test for sensitization. The filament was first pressed to the skin 1 time, after which the participant rated the pain on a HVisual Analogue Scale (VAS; 0 = No Pain, 100 = Worst Pain). This was followed by a train of 10 stimulus applications with the same von Frey filament (rate of 1 Hz), after which the participant gave a single pain rating for the series of stimuli on the VAS. For each of the three trials, the difference in pain ratings (rating for the train of 10 stimuli - rating for the single stimulus) was calculated, with sensitization defined as the mean ΔVAS on the 3 experimental trials. Positive VAS changes indicated habituation, and negative changes indicated sensitization.

### 2.4. Power Analysis

The primary comparison of interest was heat pain threshold between youth with chronic pain and normal weight (CP) and youth with chronic pain and obesity (CPO), at the hand. We planned to include 20 in each group to have at least 80% power to detect a difference of 1.1 standard deviations. Due to time restrictions, we had to terminate the study early. Nonetheless, with our obtained sample size (*n* = 12 CP, *n* = 19 CPO), we had the power to detect a difference of 1.2 standard deviations. Because of the small sample size, we used the term “significant” for an uncorrected *p* < 0.05. 

### 2.5. Statistical Analysis

The threshold and sensitization indices were skew and, therefore, between-group assessments were examined using non-parametric Kruskal–Wallis tests, with Mann–Whitney tests used for post-hoc analyses. Data are reported as median (interquartile range). Normally distributed variables are reported as mean (standard deviation). The Spearman’s rho correlation coefficient was used to analyze relationships between experimental pain and clinical pain parameters for the two groups with chronic pain. Effect size was estimated with partial eta squared (*η^2^)*. As referenced in other studies [21], effect size interpretation is as follows: *η^2^* = 0.01 indicates a small effect, *η^2^* = 0.06 a medium-sized effect and partial *η^2^* = 0.14 a large effect. All analyses were conducted with SPSS (IBM Corp, Chicago, IL, USA) v21.0. 

## 3. Results

The testing procedures were well tolerated by all participants. Despite the fact that some participants reached the maximum allowable temperature for safety (50 °C) during the HPT tests, no participants withdrew from the study. 

### 3.1. Recruitment

In total, 637 patients from both clinics were assessed for participation. Of the pain clinic sample, 211 were ineligible, primarily due to age and medication usage. Of the 73 approached for the study, 30 declined. Ultimately, 31 patients (CP = 12; CPO = 19) with chronic pain participated. Of the obesity clinic sample, most were ineligible due to age, and of the 29 approached, 7 declined. Ultimately, 14 completed the study. 

### 3.2. Participants

Participant demographics are shown in Table 1. Based on BMI percentile for age and gender [36], participants with chronic pain were categorized as underweight (BMI < 5 percentile), normal weight (BMI ≥ 5th and < 85th percentile), overweight ≥85th and < 95th percentile), or obese (≥ 95th percentile). The chronic pain (CP) group included 2 participants with underweight and 10 participants with normal weight. The chronic pain + obesity (CPO) group included 8 participants with overweight and 11 with obesity. As indicated, with the exception of appropriate differences in BMI and BMI Z-score, these participants did not differ on any of the demographic, pain, or QST variables, and were therefore combined for simplicity of data interpretation. Across the three groups, participants did not differ in age, gender or ethnicity (*p* > 0.05). 

The primary pain locations for the CP group were mainly head pain (25%) and back/chest pain (25%). The primary pain locations for the CPO group were mainly head pain (32%), generalized/myofascial pain (26%), and back/chest pain (21%). Other pain characteristics are shown in Table 1. No group differences were found on usual or worst pain intensity in the past 2 weeks, nor on duration of the pain problem. The O group reported significantly fewer days in the past 2 weeks with pain than the CPO group (*p* < 0.01). 

### 3.3. Heat Pain Threshold

**Figure 1:**
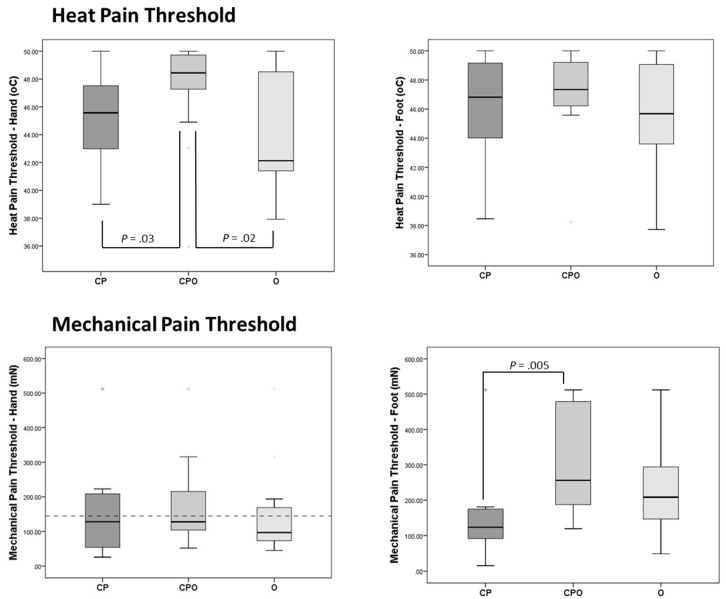
Heat and mechanical pain thresholds by weight/diagnostic group and for all participants with chronic pain.

Participants in the CPO group had significantly higher (less sensitive) heat pain thresholds at the hand than participants in the O group (*p* = 0.03) and the CP (*p* = 0.02) groups. No between-group differences were found for heat pain threshold at the foot (data shown in Table 2 and Figure 1).

### 3.4. Mechanical Pain Threshold

No between-group differences were found for mechanical pain threshold at the hand. At the foot, participants in the CPO group had significantly higher (less sensitive) mechanical pain thresholds than participants in the chronic pain group. Although the median threshold at the foot was higher in the CPO group than in the O group, the difference was not significant (data shown in Table 2 and Figure 1).

### 3.5. Heat Perceptual Sensitization

**Figure 2:**
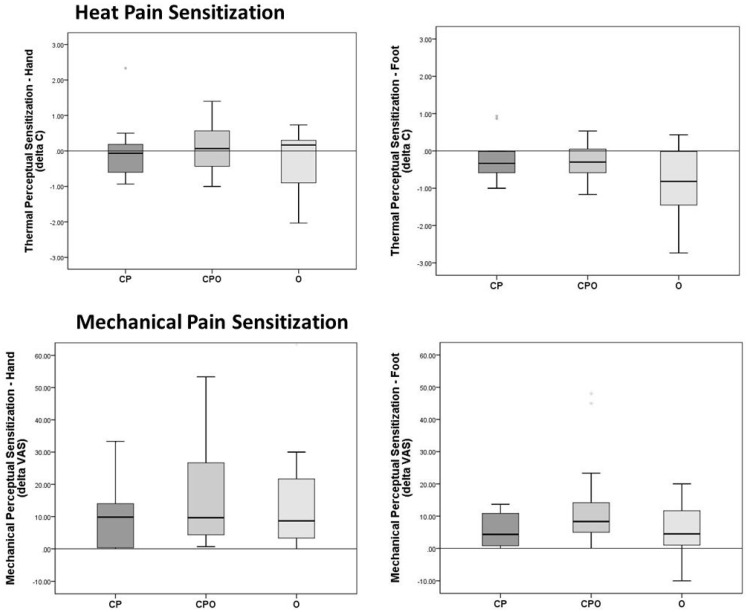
Heat (ΔT (°C), *Mdn (IQR)*) and mechanical perceptual sensitization (ΔVAS, *Mdn* (IQR)) at the hand and foot.

Negative changes in temperature or VAS pain ratings indicate sensitization (heat and mechanical tests, respectively), whereas positive changes indicate habituation. Data are shown in Table 3, Figure 2. Although no between-group differences were found at either testing site, we were unable to determine HPS at the hand and/or foot for eight participants because their pain thresholds were at or above the 50 °C cutoff for safety (i.e. the test could not be completed because the TSA-II shut down at 50 °C). Specifically, we were unable to measure the HPS for five participants at the hand and seven participants at the foot. For one additional participant (not included in the eight), we were able to measure the HPS (thenar) for two of the three experimental trials, and calculated HPS based on the two values. Of the eight participants, one was from the CP group, four were from the CPO group, and three were from the O group. Adjusted *n* values are noted in Table 3.

### 3.6. Mechanical Perceptual Sensitization

No between-group differences were found at either site (*p* > 0.05). Data are shown in Figure 2 and Table 3.

### 3.7. Correlations between Experimental Pain and Clinical Pain in Participants with Chronic Pain

CP group: No threshold or sensitization measures were correlated with any of the clinical pain measures. CPO group: Usual pain intensity was moderately negatively correlated with HPT at the foot (*r_s_* = −0.59; *p* = 0.008), and with the MPT at both the hand (*r_s_* = –0.51, *p* = 0.025) and the foot (*r_s_* = −0.64, *p* = 0.003). Worst pain intensity was moderately negatively correlated with both the MPT at the hand (*r_s_* = –0.47, *p* = 0.049) and the foot (*r_s_* = –0.49, *p* = 0.039). In sum, these correlations indicate that for participants in the CPO group only, sensitivity to experimental pain in both modalities and body sites was directly related to clinical pain intensity.

## 4. Discussion

This is the first study to examine the potential impact of obesity on sensory functioning in adolescents with chronic pain. QST was used to examine relative differences in pain thresholds and central sensitization in three groups: chronic pain (CP), obesity (O), and chronic pain + obesity (CPO). Participants in the CPO group showed significant differences in pain thresholds on two of the four tests, in different sensory modalities, and both were large effect sizes. Specifically, the heat pain threshold at the hand was significantly higher in the CPO group than in the CP and the O groups, and the mechanical pain threshold at the foot was significantly higher in the CPO group than in the CP group. Furthermore, the CP and O groups did not differ on any of the pain threshold tests. Tests of perceptual sensitization were not different across groups for either sensory modality, and the effect sizes were small. Finally, experimental pain was directly related to clinical pain for the CPO group, but experimental and clinical pain were unrelated for the CP group. Together, these results suggest that the double morbidity affects sensory processing in ways unlike that associated with either condition alone. Altered sensory functioning in any pain condition has important implications [13], which may be truer in the case of chronic pain complicated by co-occurring obesity. The current findings lay the foundation for larger investigations of the pathophysiology in youth with CPO. 

Lessons Learned: While explanations of the mechanisms underlying our findings are beyond the scope of our data, outlining lessons learned in light of the current literature offers some hypotheses, and may improve the design of subsequent studies focused on this population. 

### 4.1. Obesity Might Exacerbate the Effects of Chronic Pain on Sensory Functioning 

Simply put, differences on two of four pain threshold tests, each with a large effect size, support the plausibility of obesity negatively affecting sensory processing in youth with chronic pain. The fact that we did not find relative differences in sensitization suggests that this effect may be a local, as opposed to a central, phenomenon, consistent with an adult study [12]. If obesity does interact with chronic pain to alter sensory functioning, it would be consistent with other evidence that the two conditions interact, such that the combined effects are different from those associated with each individual condition [7,8,9,10]. For example, other work has shown that youth with CPO are twice as likely to have impaired physical functioning than youth with chronic pain, and more than six times more likely than youth with obesity alone [7]. 

Although no studies involving youth with both chronic pain and obesity exist, there is some evidence that chronic pain alone decreases heat and mechanical pain thresholds. Therefore, our findings of increased MPT and HPT in youth with CPO suggest that obesity alters sensory processing already affected by chronic pain, and in such a way as to be very different from the effects of chronic pain alone. For example, Zohsel et al. compared youth with and without migraines at pain (trigeminal) and non-pain (thenar eminence) sites [17]. Although no between-group differences were found for heat pain thresholds at either site, the migraine group did have significantly lowered MPT at both sites. Brandow et al. examined HPT and MPT at the hand and foot in youth with and without sickle cell disease (SCD) [19]. They found a significantly lowered HPT at the hand in youth with SCD, and no differences in MPT at either site. The differences in pain thresholds in these sensory modalities (heat and mechanical pain) are likely due to differences in the specific pain conditions examined, as well as in the sites used for testing. Nonetheless, chronic pain alone can alter sensory processing in both heat and mechanical modalities.

There are multiple mechanisms by which obesity may exacerbate altered sensory processing in a system already negatively impacted by chronic pain. For example, obesity is associated with deconditioning, which has been linked to increased sensitivity to pain [32]. Others have suggested the possibility of increased [24] or decreased [12] pain receptors in adipose tissue, as well as increased anti:pro-inflammatory cytokines within adipose tissue [12]. It is also possible that pain pathways may be modulated as a consequence of obesity-driven increases in adipokines and hormones interacting with the central opioid system. Examples include leptin, adiponectin, ghrelin, and oxytocin [11]. Increased levels of systemic inflammation characteristic of obesity may also result in increased sensitization of nociceptors [37]. The increased mechanical load associated with obesity may alter pain processing, particularly in the back and lower extremities, directly through increased mechanical pressures [32,37] and indirectly through altered biomechanics [37].

### 4.2. Obesity Should not Be Overlooked in Pediatric Studies of Somatosensory Functioning

Ours is among the few studies to examine obesity in a pediatric QST study, and, therefore, although preliminary, our findings contribute to current knowledge. There is evidence that pediatric pain thresholds can be influenced by sex [38], ethnicity [39], pain condition [16,17,18,19,20,21,23], age [15], and psychosocial factors [17]. Our findings suggest that obesity should be added to the list. The priority of subsequent studies should be to examine weight-related effects on sensory functioning in conditions known to be influenced by obesity, such as fibromyalgia [24,32], osteoarthritis [40,41,42], musculoskeletal pain [42,43,44,45] and headache/migraine pain [46,47]. Future work should also determine whether the general pattern of elevated sensory thresholds for youth with CPO holds for other QST methods.

### 4.3. These Preliminary Findings Warrant Follow-Up, Despite the Inconsistencies in the Literature

Currently, there is no clear understanding of whether obesity does or does not alter sensory processing. Studies have shown higher [11,28,29,30], lower [24,25,26], and no differences [12,31] when participants with obesity have been compared to healthy controls. Nonetheless, more evidence is on the side of obesity effecting some kind of alteration to sensory processing than on the side suggesting no effect at all. Additionally, although not based on standardized QST methods, the only studies involving participants with chronic pain (fibromyalgia) and co-occurring obesity have found that obesity is associated with greater pain sensitivity [24,32]. It is likely that the wide variety of testing sites, sensory modalities, and differences in QST techniques underlie the inconsistencies across studies. Even within studies, it is not uncommon to have inconsistencies across tests and modalities [48]. Our finding of higher pain thresholds in the CPO group is consistent with the QST studies that have found obesity to be related to higher (less sensitive) pain thresholds and sensitivities in adults. Considering that the current study included participants who were not taking any short- or long-acting psychotropic or pain medications, it is possible that our chronic pain groups were the least complex of our clinic pain population. This could possibly explain the lack of any difference between the O group (single morbidity) and the CP group (single morbidity) and would be consistent with studies showing no effect of obesity. For example, Stolzman et al. [31] tested pressure pain thresholds at three body sites (quadriceps, deltoid, and finger nailbed) in healthy youth with and without obesity, and found no between-group differences for any of the tests. Therefore, while there is almost nothing like the current study in the literature, findings generally support an effect of obesity, and therefore warrant follow-up.

### 4.4. As in Other Areas of Science, Overcoming Methodological Hurdles may Result in New Understanding for the Population under Study 

Obesity could easily be considered a potential confound or methodological hurdle in QST studies. Therefore, study design and QST method must be adapted to study the effects of obesity on somatosensory function. Higher pain thresholds in those with obesity have been explained, in part, as a result of moderation by thicker subcutaneous adipose tissue thought to stifle afferent sensation [12]. This hypothesis “offered” Price et al. [12] the opportunity to explore ways to examine sensory functioning while controlling for adiposity. The solution, as it were, was to conduct sensory tests on areas of the body with and without thick subcutaneous adipose tissue. These researchers assessed sensory functioning on the abdomen (thick subcutaneous adipose tissue) and on the hand and forehead (little to no adipose tissue). The finding of higher pain thresholds/less sensitivity at the abdomen but not the hand or forehead was interpreted as threshold differences likely reflecting a peripheral phenomenon, without any central involvement. Further, the authors suggested that the decreased sensitivity to pain in areas with a greater degree of adipose tissue may have been due to (1) a decreased density of nociceptors in the skin stretched over adipose tissue, and/or (2) a greater proportion of anti- to pro-inflammatory cytokines within the adipose tissue itself. Findings such as these raise critical questions, and it is the pursuit of answers to these questions that may lead to a greater understanding of pain for those who suffer from chronic pain + obesity. 

In the current study, we chose our anatomical testing sites based on Price et al.’s rationale. Nonetheless, in contrast to their findings, we found relative differences in pain thresholds, therefore adiposity cannot explain our findings. Further, although BMI is an imperfect measure of adiposity, the O group had a significantly higher median BMI percentile than the CPO group; nonetheless, the CPO group had a higher heat pain threshold at the hand. What we do not know, however, is whether sensory fiber innervation differs across the groups in this study. Future studies will need to control for potentially confounding factors such as adiposity, and should be based on standardized QST methods [15], the combination of which may not be easy. Future studies will also need to utilize multiple QST methods and modalities, to more fully examine sensory processing in this population, and to determine the mechanisms underlying observed sensory differences. Our finding of a higher mechanical pain threshold at the foot is a result of our attempt to avoid adiposity as a confound, yet it may be a potentially promising area of future research. The foot is a common pain location for youth with obesity [49], which is thought to be based primarily on excess forces related to body mass. It is widely suggested that excess body mass causes or exacerbates foot pain, which in turn may limit mobility [50,51], and ultimately negatively affect health-related quality of life. However, to our knowledge, no prior studies have examined an effect of obesity on sensory processing in the foot. 

### 4.5. The Implications Demand Continued Pursuit of Definitive Answers 

Given that youth with CPO do not benefit from multidisciplinary pain management, and they experience significantly impaired health-related quality of life in all domains, it is imperative that we continue to pursue a deeper understanding of pain for this population. It will be important for future studies to determine whether differences in pain processing are related to clinical pain. We found that, for the CPO group only, experimental pain was related to usual and worst pain intensity, as well as to duration of pain problem. These results further suggest that the combination of chronic pain + obesity is distinct from either condition alone. Our data may also suggest that, for the CPO group, it takes increased intensity to perceive a stimulus as painful, which may mean that it may take longer to report pain, and in turn, longer to be referred for pain treatment. This is also consistent with the literature [9]. If true, the increased exposure to “painful” stimuli and compounded treatment delay may explain the treatment failure observed for this population. 

### 4.6. Limitations and Strengths

The results of this study must be considered in light of limitations. First, based on the pilot nature, the sample size was small. This may have affected the power to detect between-group differences. Related to this, the study is limited by the heterogeneity of pain diagnoses represented in participants with chronic pain. Future studies would benefit from a more homogeneous sample, as well as a larger sample size. Third, while we assessed pain in O group participants, we did not exclude those with pain of more than 3 months. It is possible that data for this group was affected by pain in these participants. However, the median duration of pain for the O group was < 3 months, and, given the results of the study, it seems unlikely that any pain in this group affected participants to the same degree as those in the CPO group. Finally, given the inability to assess sensitization in some patients with obesity (due to elevated heat pain thresholds), it may be that heat pain is not an appropriate modality for the assessment of sensitization in patients with obesity. Strengths: a strength of this study is that all QST assessments were conducted by a single investigator with doctoral training in psychophysics (KH). The QST protocol was modeled after a standardized protocol used in a pediatric QST study. Additionally, QSTs in this study were unaffected by long- or short-acting pain medications. For this reason, our data are untainted by medication use, although our sample may not be representative of the larger pediatric pain population. 

## 5. Conclusions

This pilot study suggests that chronic pain and obesity may interact to increase heat and mechanical pain thresholds (decrease sensitivity) in youth with co-occurring chronic pain and obesity. While this interaction would be consistent with other studies focused on this population, future studies are warranted. 

## Figures and Tables

**Table 1:** Participant demographics and pain characteristics by group.

	CP	CPO	O	*p* Value
	(*n* = 12)	(*n* = 19)	(*n* = 14)	
**Age (years)**				
Mean (± SD)	15.3 (± 1.6)	16.0 (± 1.2)	15.1 (± 1.4)	0.21
Range	13.0–17.5	13.8–17.8	13.1–17.3	
**Race**				0.14
AA	41.70%	31.60%	71.40%	
Caucasian	50%	57.90%	21.40%	
Hispanic	8.30%	--	7.10%	
Asian	--	10.50%	--	
**Female**	58.30%	52.60%	71.40%	0.56
**BMI %ile**				
Median	52	95	99	<0.001 ^a^
(IQR)	(38.8–65.0)	(92.0–99.0)	(98.0–99.0)	
**Days with pain in past 2 weeks**	8	14	3	0.001 ^b^
	(2.8–13.3)	(7.0–14.0)	(2.0–7.0)	
**Usual Pain Intensity**	6.5	5	5.5	0.78
	(4.5–7.8)	(4.0–7.0)	(4.0–7.8)	
**Worst Pain Intensity**	8.5	8.5	7	0.4
	(7.3–10.0)	(7.8–9.3)	(6.0–9.0)	
**Duration of Pain Problem (months)**	11.5	12	2.5	0.54
	(6.3–24.0)	(5.0–28.0)	(0.0–27.0)	

^a^ All pairwise comparisons differ (*p* < 0.03–0.001). ^b^ O differed significantly from CPO (*p* < 0.001). No other differences were significant (*p* > 0.05).

**Table 2:** Heat and mechanical pain thresholds by group.

	**CP**	**CPO**	**O**	***p* Value**	***η*^2^**
	**(*n* = 12)**	**(*n* = 19)**	**(*n* = 14)**		
**Heat Pain**					
Hand	45.6 (42.5–47.9)	48.4 (46.6–49.8)	42.1 (41.4–48.7)	**0.02 ^a^**	**0.13 ^c^**
Foot	46.8 (43.9–49.6)	47.3 (46.1–49.5)	45.7 (42.7–49.1)	0.3	0.01
**Mechanical Pain**					
Hand	128.3 (51.3–215.6)	128.0 (104.0–222.9)	97.0 (72.3–175.2)	0.47	0.012
Foot	123.7 (85.1–178.0)	256.0 (181.0–512.0)	208.4 (134.5–305.0)	**0.005 ^b^**	**0.15 ^c^**

^a^ CPO significantly higher than CP (*p* = 0.03) and O (*p* = 0.02). No difference between CP and O (*p* = 0.49). ^b^ CPO significantly higher than CP (*p* = 0.005). No differences between CPO and O (*p* = 0.13), or CP and O (*p* = 0.16). ^c^ Large effect sizes are bolded.

**Table 3:** Heat (ΔT (°C), Mdn (IQR)) and mechanical perceptual sensitization (ΔVAS, *Mdn* (IQR)) at the hand and foot for each group.

	**CP**	**CPO**	**O**	***p*** **Value**	*η*²
**Heat Sensitization**	(*n* = 12) ^a^	(*n* = 17) ^a^	(*n* = 11) ^a^		
Hand	−0.1 (−0.7–0.2)	0.1 (−0.5–0.6)	0.2 (−1.0–0.4)	0.6	0.026
	(*n* = 11) ^a^	(*n* = 13) ^a^	(*n* = 15) ^a^		
Foot	−0.3 (−0.6–0.0)	−0.3 (−0.6–0.1)	−0.8 (−1.6–0.0)	0.29	0.013
**Mechanical Sensitization**	(*n* = 12)	(*n* = 19)	(*n* = 14)	0.47	
Hand	9.8 (0.2–14.3)	9.7 (4.0–26.7)	8.7 (3.3–22.9)		0.012
Foot	4.3 (0.4–11.3)	8.3 (4.7–15.0)	4.5 (0.8–12.1)	0.15	0.043

^a^ The numbers of participants for the HPS assessments reflect only participants for whom a HPS could be calculated. Adjusted *n* values are shown for each group.
